# Identification and vitro verification of the potential drug targets of active ingredients of Chonglou in the treatment of lung adenocarcinoma based on EMT-related genes

**DOI:** 10.3389/fgene.2023.1112671

**Published:** 2023-02-07

**Authors:** Fulai Zhao, Peng Zhao, Junli Chang, Xingyuan Sun, Xiaoping Ma, Binhao Shi, Mengchen Yin, Yongjun Wang, Yanping Yang

**Affiliations:** ^1^ Longhua Hospital, Shanghai University of Traditional Chinese Medicine, Shanghai, China; ^2^ Key Laboratory of Theory and Therapy of Muscles and Bones, Ministry of Education, Shanghai, China

**Keywords:** lung adenocarcinoma, Epithelial-Mesenchymal Transition, drug targets, active ingredients in Chonglou, bioinformatics, network pharmacology

## Abstract

Lung adenocarcinoma (LUAD) is the main histological type of lung cancer with an unfavorable survival rate. Metastasis is the leading LUAD-related death with Epithelial-Mesenchymal Transition (EMT) playing an essential role. The anticancer efficacies of the active ingredients in Chonglou have been widely reported in various cancers. However, the potential therapeutic targets of the Chonglou active ingredients in LUAD patients remain unknown. Here, the network pharmacology and bioinformatics were performed to analyze the associations of the clinical characteristics, immune infiltration factors and m^6^A-related genes with the EMT-related genes associated with LUAD (EMT-LUAD related genes), and the molecular docking, STRING, GO, and KEGG enrichment for the drug targets of Chonglou active ingredients associated with EMT (EMT-LUAD-Chonglou related genes). And, cell viability analysis and cell invasion and infiltration analysis were used to confirm the theoretical basis of this study. A total of 166 EMT-LUAD related genes were identified and a multivariate Cox proportional hazards regression model with a favorable predictive accuracy was constructed. Meanwhile, the immune cell infiltration, immune cell subsets, checkpoint inhibitors and the expression of m^6^A-related genes were significantly associated with the risk scores for EMT-LUAD related genes with independent significant prognostic value of all included LUAD patients. Furthermore, 12 EMT-LUAD-Chonglou related genes with five core drug targets were identified, which participated in LUAD development through extracellular matrix disassembly, collagen metabolic process, collagen catabolic process, extracellular matrix organization, extracellular structure organization and inflammatory response. Moreover, we found that the active ingredients of Chonglou could indeed inhibit the progression of lung adenocarcinoma cells. These results are oriented towards EMT-related genes to achieve a better understanding of the role of Chonglou and its targets in osteosarcoma development and metastasis, thus guiding future preclinical studies and facilitating clinical translation of LUAD treatment.

## Introduction

Lung cancer is the most commonly occurring cancer and the leading cancer-related death accounting for 18.0% of the total cancer deaths. Though multimodality personalized treatments have been developed, the 5-year overall survival rate of lung cancer patients is only 10%–20% across different regions and counties (Sung et al., 2021). Almost 40% of all lung cancer is the lung adenocarcinoma (LUAD) (Ramadori et al., 2015). Metastasis is the major trigger of LUAD-related death (Pang et al., 2017) which is widely accepted to be closely associated with the Epithelial-Mesenchymal Transition (EMT). As an essential biological process for embryonic development (Kalluri and Weinberg, 2009), EMT has been well known to promote the occurrence, metastasis and drug resistance of cancers (Du and Shim, 2016; Pastushenko and Blanpain, 2019). During EMT process, epithelial cell markers are downregulated while mesenchymal cell markers are upregulated, resulting in decreased cell adhesion and increased malignant features of the cancer cells, such as the metastases (Bilyk et al., 2017; Dongre and Weinberg, 2019; Wang et al., 2021). Furthermore, EMT has been demonstrated to be the poor prognostic marker in LUAD patients (Soltermann et al., 2008; Tischler et al., 2011). Therefore, the EMT-related genes associated with LUAD (EMT-LUAD related genes) are important molecular biomarkers in new therapeutics exploration for LUAD management.

Chonglou, known as Paris polyphylla Smithvar. Yunnanensis (Franch.) Hand.-Mazz, or P. polyphylla Smithvar. Chinensis (Franch.), is a natural medicine. The active ingredients of Chonglou have been reported to effectively inhibit various cancers, such as Dioscin and Gracillin promote the apoptosis and cell cycle arrest in lung cancer, colorectal cancer and osteosarcoma cells (Ding et al., 2020; Wu et al., 2020; Yang J. et al., 2021) Meanwhile, polyphyllin Ⅰ inhibits osteosarcoma development through inactivating Wnt signaling pathway (Chang et al., 2017), and polyphyllin Ⅱ inhibits the metastasis of bladder cancer cells by regulating EMT (Niu et al., 2020). Chonglou is a traditional Chinese anti-tumour herbal medicine and is often used as one of the ingredients in many patented Chinese anti-tumour drugs in modern clinical treatment to reduce toxicity and increase the efficiency of treatment, such as Gan-Fu-Le capsules for the adjuvant treatment of primary hepatocellular carcinoma and Bo-Er-Ning capsules for the adjuvant treatment in many cancers (Wang et al., 2018). However, the drug targets and the potential therapeutic mechanisms of the Chonglou active ingredients in LUAD management need to be further explored. Therefore, it is necessary to explore the relationship between the Chonglou active ingredients and the EMT-LUAD related genes, thus to identify the new potential drug targets for improving the poor prognosis of LUAD patients. Meanwhile, with the development of high-throughput sequencing and analysis technologies, the opportunity of comprehensively understanding the cancer genome to identify the key cancer-related genes as drug targets has become possible (Lee et al., 2012).

In this study, we intended to investigate the potential drugs of Chonglou active ingredients for LUAD based on EMT-related genes by using the bioinformatics and network pharmacology methods, and to clarify the possible mechanisms of Chonglou active ingredients for LUAD treatment, which will provide new strategies for LUAD management.

## Methods and materials

### Identification of the EMT-related genes in the oncogenesis and development of LUAD patients

To explore the EMT-related genes, which are associated with the oncogenesis and development of LUAD, transcriptome profiles of LUAD patients were obtained from The Cancer Genome Atlas (TCGA) database (https://portal.gdc.cancer.gov/). The “limma” package of R-language Bioconductor was used to identify the differentially expressed genes (DEGs) of LUAD patients under the conditions of false discovery rate <0.05 and |log(FC)| ≥ 4. The EMT-related genes were respectively extracted from the Genecard database (https://www.genecards.org/), the OMIM database (https://omim.org/), the EMTome (http://www.emtome.org/), the Epithelial-Mesenchymal Transition Gene Database (http://dbemt.bioinfo-minzhao.org/index.html), and the NCBI (https://www.ncbi.nlm.nih.gov/). Then the overlapping genes between the EMT-related genes and the LUAD associated genes (EMT-LUAD related genes) were identified with the Venn diagram and used for the following analysis (Wu et al., 2019; Li et al., 2020).

### Correlation analysis between the EMT-LUAD related genes and the clinical characteristics of LUAD patients

The ‘survival’ package in R-language was used to analyze the correlation between the EMT-LUAD related genes and the survival of LUAD patients.

First, to identify the EMT-LUAD related genes with significant prognostic value (*p* < .05), the univariate Cox proportional hazards regression analysis was used, and the correlation between the EMT-LUAD related genes and the survival of LUAD patients was analyzed.

Next, to identify EMT-LUAD related genes with independent significant prognostic value, a multivariate Cox proportional hazards regression model was constructed. The EMT-LUAD related genes with independent significant prognostic value was further analyzed (the mode of stepwise search is “both”). As a result, the risk scores for all EMT-LUAD related genes with independent significant prognostic value of each included individual were obtained [predict.coxph (type = “risk”) of “survival” package in R language], and the median risk score was used as the cut off value to divide the LUAD patients into two subgroups, including a high-risk score group and a low-risk score group.

Then, the expression heatmap of EMT-LUAD related genes with independent significant prognostic value in the high- and low-risk groups was plotted in R language using the package “pheatmap”. Meanwhile, the risk score curves and the patient survival scatter plots of all included individuals, the survival curves of the high- and low-risk groups, and the time-dependent ROC curves of this model were carried out in R-language with the package “survival” and “survminer”.

Last, the association between the EMT-LUAD related genes with independent significant prognostic value and the clinical characteristics were analyzed in R-language with the package “survival”, including the [age (≤60 *versus* >60), gender (male *versus* female), stage (stage I and II *versus* stage III and IV), T (T1 and 2 *versus* T3 and 4) and N(N0 *versus* N1–3)] (Fisher and Lin, 1999).

### Correlation analysis between the EMT-LUAD related genes and the immune infiltration factors in LUAD patients

The TIMER, CIBERSORT, CIBERSORT-ABS, and QUANTISEQ algorithms were used to explore the cellular immune response between the low-risk group and the high-risk group mentioned above.

Meanwhile, the differences of the cellular immune response between different algorithms were visualized with the Heatmap.

Furthermore, ssGSEA was used to quantify the subgroups of the tumor-infiltrating immune cells, the immune check-points, as well as the immune functions between the low-risk group and the high-risk group (Tang et al., 2021).

### Correlation analysis between N6-methyladenosine (m6A)-related genes and EMT-LUAD related gene based risk scores

The expression differences of m^6^A-related genes, including ALKBH5, HNRNPC, METTL14, METTL3, YTHDF2, IGF2BP1, IGF2BP3, METTL16, RBMX, IGF2BP2, and HNRNPA2BA, were analyzed between the low-risk and the high-risk groups mentioned above in LUAD patients (Huang et al., 2020).

### Identification of key drug targets from the active ingredients in Chonglou based on EMT-LUAD related genes

Drug targets of the active ingredients in Chonglou (dioscin (Tao et al., 2018; Ding et al., 2020; Wu et al., 2020), gracillin (Liu et al., 2021; Yang J. et al., 2021; Yang L. et al., 2021), polyphyllin I (Chang et al., 2017; Tian et al., 2020; Lai et al., 2021; Shen et al., 2021), polyphyllin II (Niu et al., 2020), polyphyllin III (Zhou et al., 2021), polyphyllin VI (Liu et al., 2020; Teng et al., 2020), polyphyllin VII (He et al., 2020; Zhao et al., 2021) and polyphyllin H) were collected by using the online tools (Su et al., 2019), including PubChem (https://pubchem.ncbi.nlm.nih.gov/), Swiss Target Prediction (http://www.swisstargetprediction.ch/index.php) and TargetNet (http://targetnet.scbdd.com/home/index/).

The overlapping gene set between the drug targets of Chonglou active ingredients with the EMT-LUAD related genes were enquired in the STRING database (version 11.5) (EMT-LUAD-Chonglou related genes), which was used for the following protein-protein interactions (PPI) network, hub genes and enrichment analyses.

### Protein-protein interaction (PPI) network and hub gene analyses for the EMT-LUAD-Chonglou related genes

The protein-protein interaction (PPI) network map of the EMT-LUAD-Chonglou related genes was further obtained by using the online tool STRING database (version 11.5). Cytoscape software was then applied to analyze the degree values, and the top five genes were further identified as hub genes with the cytoHubba plugin (Li et al., 2021).

### Enrichment analysis and network diagram construction for the EMT-LUAD-Chonglou related genes

To explore the gene biological process of the EMT-LUAD-Chonglou related genes identified above, the package ggplot2 in R software was used to perform the gene ontology (GO), and the Kyoto Encyclopedia of Genes and Genomes (KEGG) pathway enrichment analyses (Li et al., 2019; Liang et al., 2020).

Furthermore, a graphical representation of the drug-disease-target-GO function-pathway was constructed by using the Cytoscape software to create the GO and KEGG pathway analyses (Xu et al., 2021).

### Molecular docking

To carry out the molecular docking between the active ingredients of Chonglou with the five hub genes, the protein structures were obtained from the PDB database (https://www.rcsb.org/), and the compound structures were downloaded from the PubChem database (https://pubchem.ncbi.nlm.nih.gov/).

After setting the key parameters, such as the corresponding protein, the hydrogenation and the Gasteiger charge for merging nonpolar hydrogen atoms (Trott and Olson, 2010), the AutoDock Tools (version 1.5.7) were then used for the molecular docking to obtain the groups presenting the lowest protein binding energy with a rigid macromolecule as the protein structure and a genetic algorithm as the algorithm.

PyMOL (version 2.5) software was used to visualize the docking results.

### Cell incubation

The PC-9 cell lines present in this study were obtained from Nation Collection of Authenticated Cell Cultures. All cells were cultured in a 5% CO2 humidified incubator with a growth medium including FBS (10%), streptomycin (100 μg/ml) and penicillin (100 U/ml). Human lung adenocarcinoma cells were cultured at 37°C with DMEM.

### Determination of active ingredient concentration in follow-up validation

After reviewing the literature we identified Dioscin and polyphyllin III as the same compound with the same CAS number, but with different spatial structures. Finally, We determined the drug concentrations of these seven active ingredients of Chonglou. Among them, the concentration of polyphyllin I, Polyphyllin II, Polyphyllin VII and Polyphyllin H was 1 μM, the concentration of Gracillin was 2 μM, the concentration of Polyphyllin VI was 32 μM, and the concentration of Polyphyllin III was 4 μM.

### Cell viability assay

PC-9 cells (4×10^4^/100μl/well) plated in 96-well plate were placed in 96-well plates and treated with 2 μM of Gracillin, 1 μM of Polyphyllin I, 1 μM of Polyphyllin II, 4 μM of Polyphyllin III, 32 μM of Polyphyllin VI, 1 μM of Polyphyllin VII and 1 μM of Polyphyllin H. Cell viability was then measured using the Cell Counting Kit-8 (CCK-8) to determine cell viability at 24, 48, and 72 h.

Cell viability of human lung adenocarcinoma (PC-9) cells (3×10^3^/100 μl/well) plated in specific xCELLigence-E plates was sequentially and in real time monitored for 72 h using the xCELLigence-RealTime Cell Analyzer (Roche Applied Science, Mannheim, Germany).

### Transwell migration assay

PC-9 cells were cultured for 24 h in the upper chamber of a 24-well plate (3×10^4^/well/200 µl of serum-free culture medium) and the lower chamber was chemotactic culture medium (10% FBS, 300 µl). Cells were migrated to the other side of the chamber, washed lightly twice with PBS, fixed in 4% paraformaldehyde for 30 min, coloured with 1% crystal violet solution for 10 min as well as photographed under the microscope.

### Real time migration and invasion assay

The xCELLigence RTCA DP system was applied to assess PC-9 cell invasion and migration. Briefly, for the specific CIM-Plate in the xCELLigence RTCA DP system, the lower chamber contained medium with 10% FBS (165 μl/well). 40 μl/serum-free medium were used in the upper chamber to determine background. Cells in serum-free medium (3 × 10^4^/100 μl/well) were then placed in the upper chamber. Cell migration capacity was monitored continuously for 24 h. For the cell invasion capacity assay, with the exception of applying the upper chamber pre-coated with Matrigel and monitored continuously for 48 h, the same conditions as for the migration assay were used.

## Results

### A total of 166 EMT-related genes are identified in LUAD development

**FIGURE 1:**
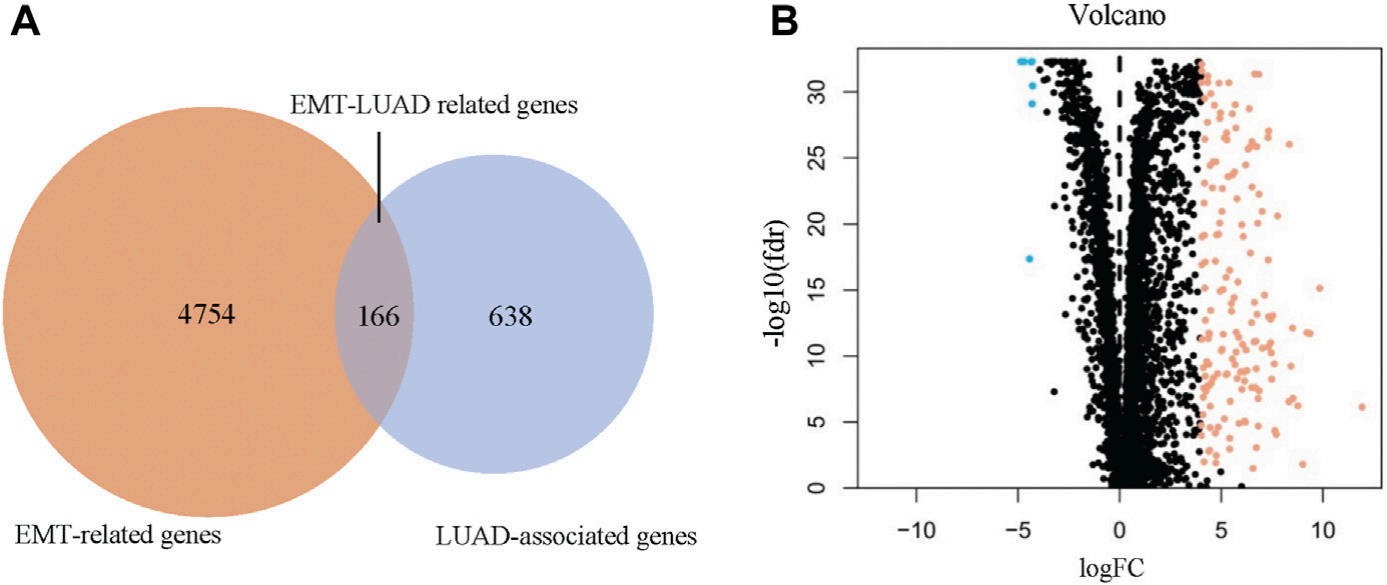
Identification of the overlapping genes between the EMT-related genes and the LUAD associated genes (EMT-LUAD related genes). **(A)** Venn diagram confirmed the EMT-LUAD related genes. **(B)** Volcano-plot of EMT-LUAD related genes (the blue dots represent the down-regulated genes and the orange dots represent the up-regulated genes).

After a comprehensive search from the public database, 4920 EMT-related genes were collected from Genecard, OMIM, and EMTome databases (Figure 1A, left circle), meanwhile, 804 DEGs between the LUAD and the normal control tissues were identified from TCGA database under the conditions of false discovery rate <0.05 and **|**log(FC)**|** ≥ 4 (Figure 1A, right circle). A total of 166 overlapping genes were recognized as the EMT-LUAD related genes (Figure1A, overlapping part), including 7 down-regulated and 159 up-regulated genes in the LUAD tissues *versus* the normal control tissues from LUAD patients (Figure 1B).

### Sixteen EMT-LUAD related genes are identified to be important prognostic factor of LUAD patients

**FIGURE 2:**
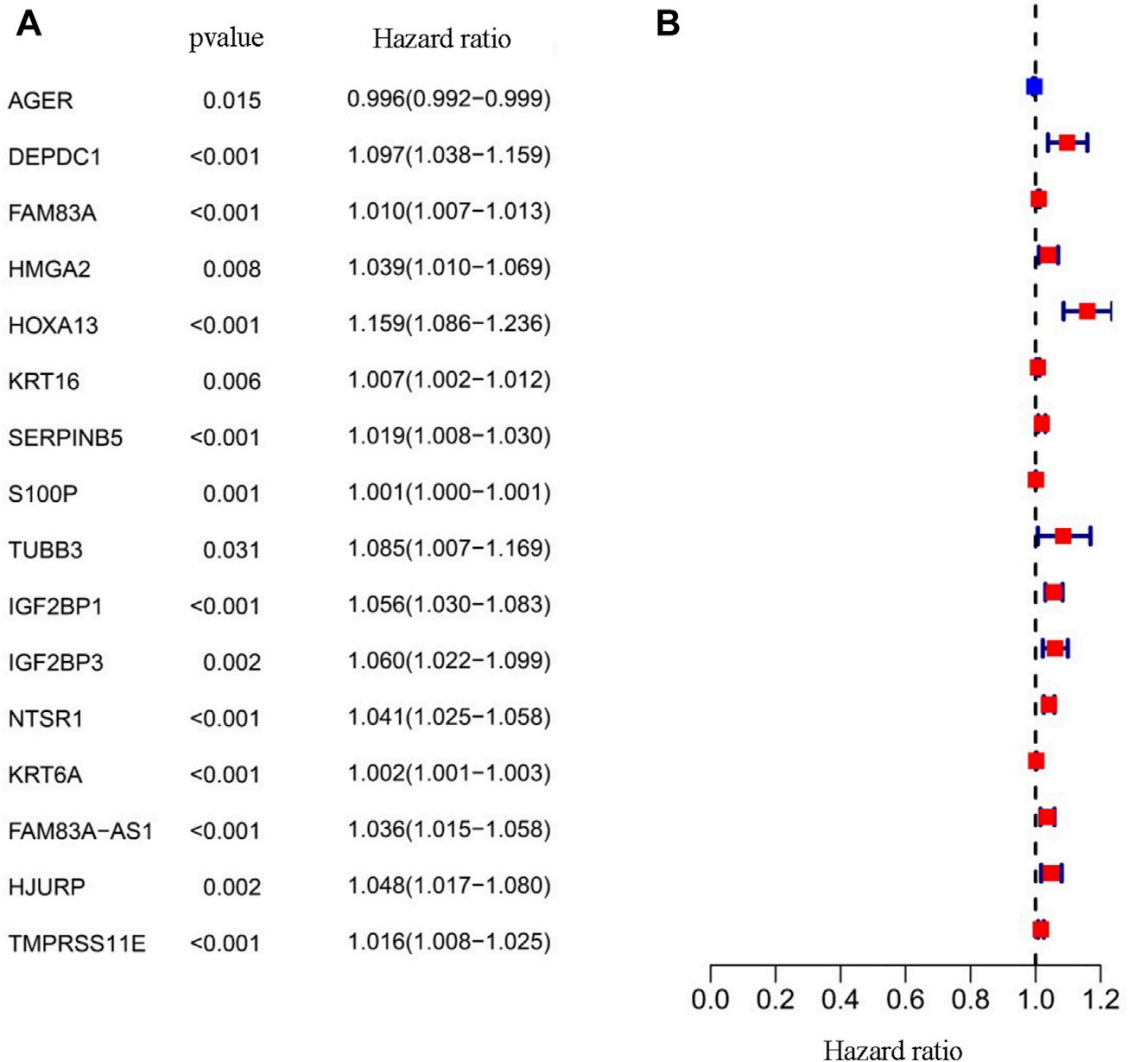
Prognostic value of the EMT-LUAD related genes. **(A)** The Univariate Cox analysis identified 16 genes, including AGER, DEPD1, FAM83A, HMGA2, HOXA13, KRT16, SERPINB5, S100P, TUBB3, IGFBP1, IGFBP3, NTSR1, KRT6A, FAM83A-AS1, HJURP, and TMPRSS11E. **(B)** The forest map showing the hazard ratio of the sixteen EMT-LUAD related genes.

As a statistical method applicable to exploring the prognostic value of a single variable, the univariate Cox proportional hazards regression analysis was used to identify the potential association between the 166 EMT-LUAD related genes obtained above with the overall survival (OS) of LUAD patients. A total of 16 genes (AGER, DEPDC1, FAM83A, HMGA2, HOXA13, KRT16, SERPINB5, S100P, TUBB3, IGF2BP1, IGF2BP3, NTSR1, KRT6A, FAM83A-AS1, HJURP, and TMPRSS11E) were identified to be significantly associated with the OS of LUAD patients (Table 1 and Figure 2, *p* < 0.05), indicating the important prognostic values of these genes in LUAD patients.

**TABLE 1:** Significant prognostic value of EMT-LUAD related genes for the survival of LUAD patients by univariate Cox proportional hazards regression analysis.

Gene	Hazard ratio (HR)	Hazard ratio of 95% confidence intervals (hr 95%CI)		*p*-value
		Upper limit (U)	Lower limit (L)	
AGER	0.995568	0.992006	0.999142	0.015125
DEPDC1	1.097144	1.038262	1.159365	0.000987
FAM83A	1.00961	1.006543	1.012686	7.23E-10
HMGA2	1.039077	1.00992	1.069077	0.008298
HOXA13	1.158567	1.085552	1.236494	9.35E-06
KRT16	1.007129	1.002084	1.012199	0.005565
SERPINB5	1.019051	1.007927	1.030298	0.000752
S100P	1.000524	1.000204	1.000844	0.001341
TUBB3	1.084921	1.007308	1.168515	0.03138
IGF2BP1	1.05582	1.029767	1.082531	2.04E-05
IGF2BP3	1.059803	1.021624	1.099408	0.001917
NTSR1	1.041314	1.024883	1.058008	6.07E-07
KRT6A	1.001981	1.001169	1.002794	1.72E-06
FAM83A-AS1	1.03625	1.015421	1.057507	0.000588
HJURP	1.048164	1.017484	1.07977	0.001913
TMPRSS11E	1.016372	1.007926	1.024889	0.000137

### Eight EMT-LUAD related genes are identified to be independent prognostic factor of LUAD patients by the prediction model was constructed

Since the existing limitation of univariate Cox proportional hazards regression analysis, it is not applicable to identify whether a particular factor is an independent prognostic factor. Therefore, the multivariate Cox proportional hazards regression model was constructed, including correlation coefficient and hazard ratio.

As a descriptive statistic, the correlation coefficient is a bivariate statistic to summarize the association between two variables, which uses a number between −1 and 1 to represent the strength and direction of an association between variables, such as to describe how a specific predictor factor affects the survival of patients. The absolute value reflects the correlation strength, and the positive or negative value reflect a positive or negative correlation (Schober et al., 2018). The hazard ratio reflects how a predictor factor affects the survival (Trkulja and Hrabač, 2019). For each of the sixteen EMT-LUAD related genes with important prognostic value in LUAD patients identified by the above univariate Cox proportional hazards regression analysis, the correlation coefficient and the hazard ratio was calculated. Eight genes, including IGF2BP1, HOXA13, NTSR1, KRT6A, TMPRSS11E, AGER, FAM83A, and FAM83A-AS1, were identified to be independently and significantly associated with the prognosis of LUAD patients (Table 2). Among them, AGER and FAM83A-AS1 were favorable prognostic factors (hazard ratio <1), and others (FAM83A, HOXA13, IGF2BP1, NTSR1, KRT6A, and TMPRSS11E) were harmful prognostic factors (hazard ratio >1).

**TABLE 2:** Independent significant prognostic value of EMT-LUAD related genes for the survival of LUAD patients by multivariate Cox proportional hazards regression analysis.

Gene	Correlation coefficient (Coef)	Hazard ratio (HR)	Hazard ratio of 95% confidence intervals (hr 95%CI)		*p*-value
			Upper limit (U)	Lower limit (L)	
AGER	−0.00264	0.997361	0.994098	1.000635	0.114005
FAM83A	0.013231	1.013319	1.007821	1.018846	1.87E-06
HOXA13	0.122277	1.130068	1.046974	1.219756	0.001701
IGF2BP1	0.043871	1.044848	1.014627	1.075969	0.003394
NTSR1	0.03044	1.030908	1.012661	1.049484	0.000835
KRT6A	0.001022	1.001023	0.999947	1.002099	0.062296
FAM83A-AS1	−0.0495	0.951709	0.912127	0.99301	0.022396
TMPRSS11E	0.015022	1.015135	1.005782	1.024576	0.001469

To further investigate the association between the expression levels of these eight genes with the prognosis of LUAD patients, the risk scores for these eight genes were obtained to calculate the median risk score, which was used as the cut off value to divide the 477 LUAD patients into a high-risk score group (*n* = 237) and a low-risk score group (*n* = 240). Then different clinical characteristics of LUAD patients were analyzed based on the high- and low-risk scores.

**FIGURE 3:**
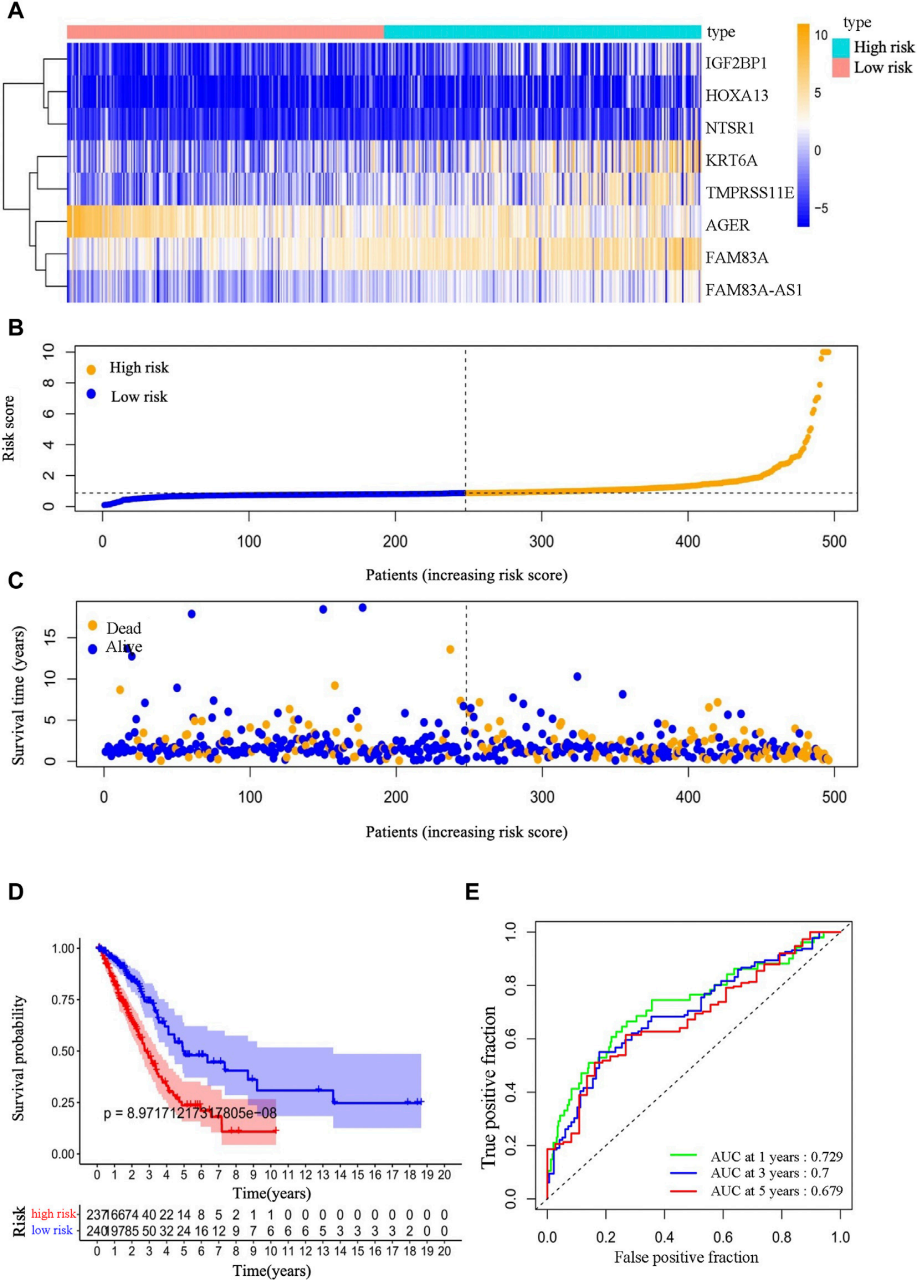
Prognostic values of EMT-LUAD related genes in our prediction model for LUAD patients. **(A)** Expression difference heatmap of the eight EMT-LUAD related genes with independent significant prognostic value for all individuals in the high- and low-risk groups. **(B)** Risk score curve of all included LUAD patients. **(C)** Survival status of all included LUAD patients [In **(B,C)**, the scatter represents an individual; the *x*-axis ranks individuals according to the risk scores from highest to lowest; the dotted line in the center of the plot represents the individuals with the median risk score]. **(D)** Survival curves of the high- and low-risk groups. **(E)** 1-year, 3-year and 5-year time-dependent ROC curve of our prediction mode.

The expression differences of the eight EMT-LUAD related genes with independent significant prognostic value between the high-group and the low-risk group were visualized by a Heatmap (Figure 3A).

The curve of risk score and the survival status of the patients were displayed (Figures 3A–C), which showed a difference between the high-risk group and the low-risk group in the survival rate, and patients in the high-risk group had a shorter overall survival than patients in the low-risk group (about 25% vs. 50% of the 5-year survival rate, *p* < 0.001) (Figure 3D). The AUC values of the ROC curves for the 1, 3, and 5-year overall survival were .729, .7, and .679, respectively, indicating a favorable predictive accuracy of the prognostic signature (Figure 3E).

**FIGURE 4:**
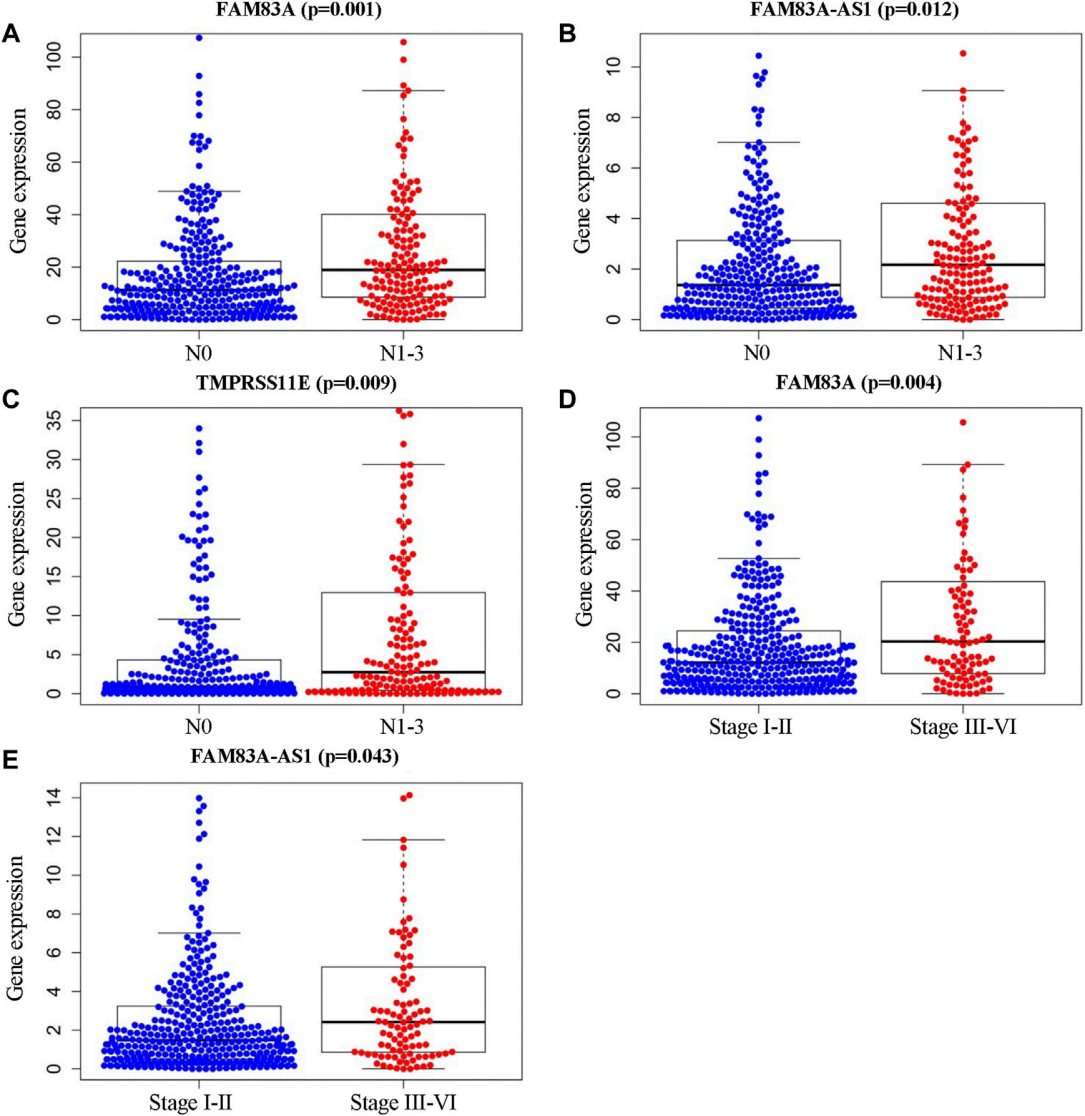
Clinical characteristic analysis of EMT-LUAD related genes with independent significant prognostic value. **(A–C)** Association of gene expression levels of FAM83A, FAM83A-AS1, and TMPRSS11E with the number of lymph node metastasis. **(D, E)** Association of FAM83A, and FAM83A-AS1 expressions with tumor stages and metastasis in LUAD patients.

Furthermore, the clinical characteristic analysis of the eight genes showed that the expression levels of FAM83A, FAM83A-AS1, and TMPRSS11E were significantly related (*p* < .05) to the number and scope of lymph node metastasis and the spread in the lymph node area, which showed that theses the expression of these three genes in the N1-3 status is higher than those in the N0 status. Moreover, FAM83A and FAM83A-AS1 were up-regulated in the stage III-IV patients than in the stage I-II patients (Table 3 and Figure 4), while AGER, HOXA13, IGF2BP1, IGF2BP1, and KRT6A were not significantly related to the development of LUAD.)

**TABLE 3:** Clinical correlation analysis of EMT-LUAD related genes with independent significant prognostic value.

Gene	Age (≤60 *versus* >60)	Gender (male *versus* female)	Stage (stage I and II *versus* stage III and IV)	T (T1 and 2 *versus* T3 and 4)	N(N0 *versus* N1–3)
AGER	−0.024 (0.981)	1.601 (0.110)	0.712 (0.478)	−0.046 (0.964)	1.718 (0.087)
FAM83A	1.193 (0.234)	−0.627 (0.531)	−2.917 (0.004)	−1.494 (0.140)	−3.322 (0.001)
HOXA13	−1.178 (0.240)	0.491 (0.624)	−1.207 (0.230)	−0.314 (0.754)	0.221 (0.825)
IGF2BP1	0.434 (0.665)	−1.706 (0.089)	−1.874 (0.063)	−1.14 (0.259)	−0.619 (0.537)
NTSR1	1.289 (0.199)	−0.588 (0.557)	−1.028 (0.306)	−1.46 (0.149)	−0.551 (0.582)
KRT6A	0.102 (0.919)	0.947 (0.344)	−0.735 (0.464)	−1.047 (0.299)	−0.755 (0.451)
FAM83A-AS1	1.687 (0.093)	−0.592 (0.554)	−2.048 (0.043)	−1.041 (0.301)	−2.518 (0.012)
TMPRSS11E	0.715 (0.475)	0.061 (0.952)	−1.237 (0.218)	−1.331 (0.188)	−2.649 (0.009)
Risk Score	0.81 (0.419)	1.392 (0.165)	−1.744 (0.084)	−1.063 (0.292)	−0.874 (0.383)

### The immune cell infiltration, immune cell subsets and checkpoint inhibitors are significantly different in the high- and the low-risk groups

**FIGURE 5:**
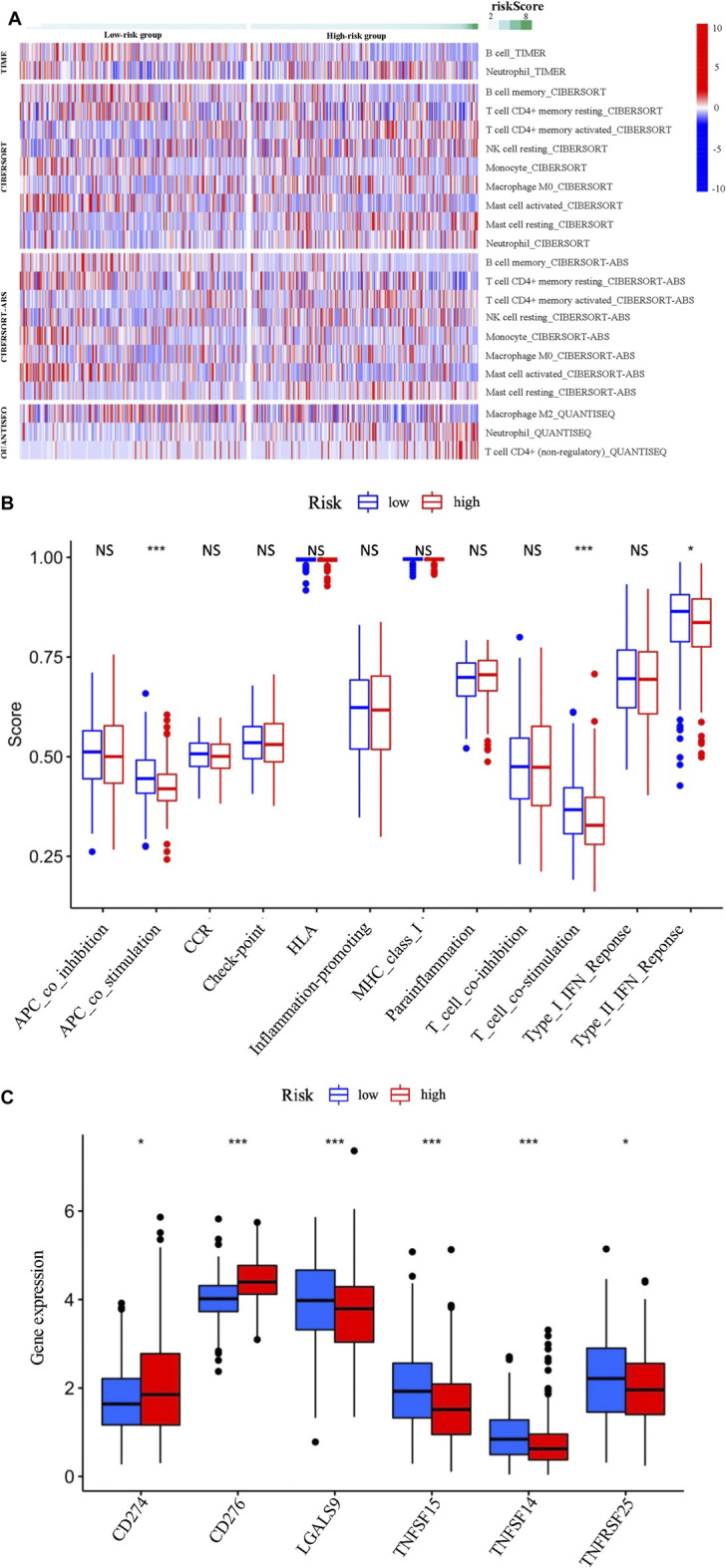
(Continued).

Since the immune system plays an important role in the oncogenesis and development of LUAD (Anichini et al., 2020), it is helpful for the clinical practice to explore the immunological regulatory effect of the EMT-LUAD related genes with independent prognostic values. Therefore, the correlation analysis between the immune cell infiltration with the risk scores of the eight EMT-LUAD related genes with independent prognostic values in the LUAD patients were further performed. And the immune responses calculated by using the TIMER, CIBERSORT, CIBERSORT-ABS, and QUANTISEQ algorithms between the high-risk group and the low-risk group were visualized with Heatmap, which revealed the correlation between the immune cell infiltration and the EMT-LUAD related genes with independent prognostic values. As shown in Figure 5A, immune cells were identified to be characteristically enriched in the low group or the high-risk groups, such as B cells calculated by TIMER, CIBERSORT and CIBERSORT-ABS, monocytes calculated by CIBERSORT and CIBERSORT-ABS, mast cells activated calculated by CIBERSORT and CIBERSORT-ABS and T cell CD4^+^ memory resting calculated by CIBERSORT-ABS being significantly enriched in the low-risk group, while neutrophils calculated by CIBERSORT and QUANTISEQ, T cell CD4^+^ memory activated calculated by CIBERSORT and CIBERSORT-ABS, mast cell resting calculated by CIBERSORT and CIBERSORT-ABS, macrophage M0 calculated by CIBERSORT and T cell CD4^+^ (non-regulatory) calculated by QUANTISEQ being significantly enriched in the high-risk group, indicating the close association between the immune infiltrations and the risk scores of the eight EMT-LUAD related genes with independent prognostic values in the LUAD patients.

Furthermore, the correlation analysis between the immune cell subsets/functions and the risk scores of the eight EMT-LUAD related genes with independent prognostic values in the LUAD patient was performed, which showed that APC_co_stimulation, T_cell_co-stimulation, and type II INF response was significantly lower in the high-than in the low-risk group (Figure 5B).

Since the checkpoint inhibitor is one of the most applicable immunotherapy strategy (Waldman et al., 2020), to identify the association between the immune checkpoints with the risks of the LUAD patients has important implications for the development of immunotherapy. Our results displayed a significant difference in the expression of CD274, CD176, LGALS9, TNFSF15, TNFSF14, and TNFSF25 between the high- and the low-risk groups. Among them, the CD274 and CD276 were downregulated, while the LGALS9, TNFSF15, TNFSF14, and TNFSF25 were upregulated in the high-risk group than in the low-risk group (Figure 5C).

### Nine m6A-related genes are identified to be significantly different between the high- and the low-risk groups of LUAD patients

**FIGURE 6:**
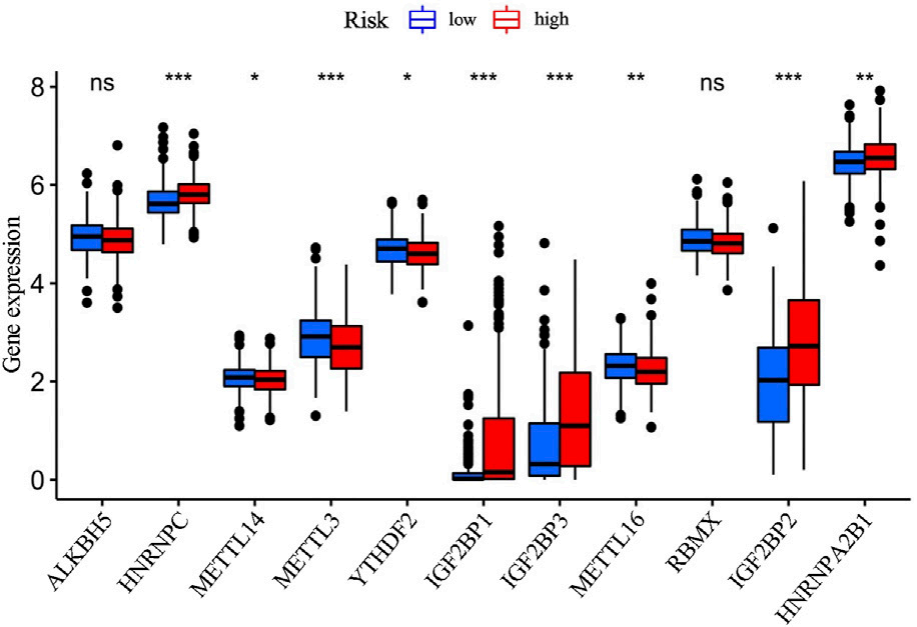
The comparative analysis of the m6A-related gene expressions between the high- and the low-risk groups.

M^6^A-related genes are important regulators in cancer development, and affect the pathological process of various cancers (Huang et al., 2020). Further comparative analysis of m^6^A-related genes between the high-risk group and the low-risk group mentioned above revealed significant differences in HNRNPC, METTL14, METTL3, YTHDF2, IGF2BP1, IGF2BP2, IGF2BP3, METTL16, and HNRNPA2B1. Among them, the expressions of HNRNPC, IGF2BP1, IGF2BP3, IGF2BP2, and HNRNPA2B1 were upregulated in the high-risk group than in the low-risk group, while the others (METTL14, METTL3, YTHDF2, and METTL16) were downregulated in the high-risk group than in the low-risk group (Figure 6).

### Twelve EMT-LUAD related genes are identified to be the drug targets of Chonglou active ingredients

**FIGURE 7:**
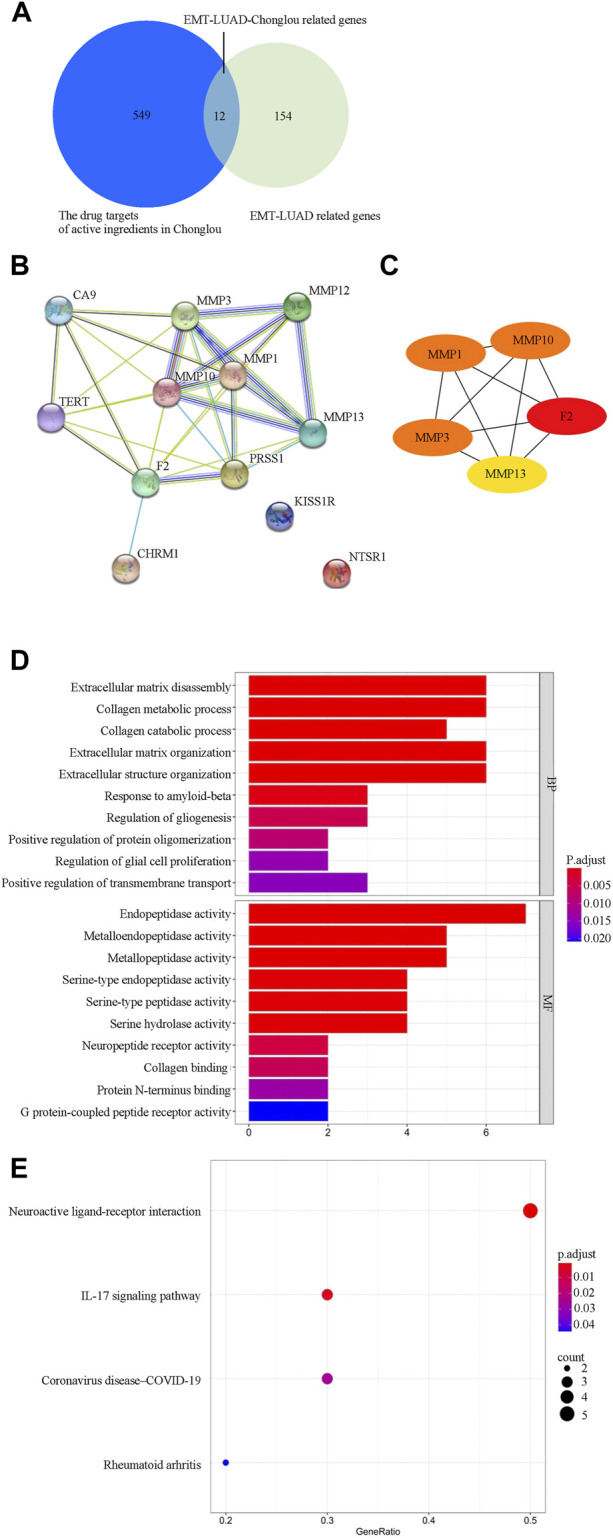
Gene network and functional characterization analysis of EMT-LUAD-Chonglou related genes. **(A)** Venn diagram depicts the cross section of the key drug targets of active ingredients in Chonglou and EMT-LUAD related genes. **(B)** STRING analysis indicating protein–protein interaction networking mediated by the EMT-LUAD-Chonglou related genes (The nodes represent proteins, the node labels are the protein names, and the connecting lines between the nodes indicate the existence of interactions between the proteins). **(C)** Cytoscape analysis representing the protein interaction network. Five core targets (F2, MMP1, MMP3, MMP10, and MMP13) are highlighted. **(D)** Gene ontology analysis of EMT-LUAD-Chonglou related genes. **(E)** Kyoto Encyclopedia of Genes and Genomes (KEGG) pathway of EMT-LUAD-Chonglou related genes.

Since the EMT-related genes play important roles in LUAD and the active ingredient of Chonglou was widely used in the treatment of various cancers, it is necessary to explore the potential drug targets of the Chonglou active ingredients from the EMT-LUAD related genes (EMT-LUAD-Chonglou related genes). Therefore, Swiss Target Prediction and TargetNet database were used for data extraction and 561 drug targets of Chonglou active ingredients were obtained (Figure 7A, left circle). Then the EMT-LUAD-Chonglou related genes were further identified by Venn diagram from the 561 drug targets of Chonglou active ingredients and the 166 EMT-LUAD related genes (Figure 7A, right circle), and 12 EMT-LUAD-Chonglou related genes were identified (Figure 7A, the overlapping part).

### Five core targets were identified from the PPI network

PPI network is used to identify the proteins based on their interaction with each other to participate in the life processes such as the biological signaling, the gene expression regulation, the energy and substance metabolism, and the cell cycle regulation (Cafarelli et al., 2017). By STRING analysis, we identified that the PPI network consisting of 12 intersection targets of EMT-LUAD-Chonglou related genes (Figure 7B). Then, the Cytoscape was further used to identify the five core targets by degree value, and F2, MMP1, MMP3, MMP10, and MMP13 were identified (Figure 7C), which suggests that these five core targets play crucial roles in the LUAD development by regulation of other genes.

### Biological pathways associated with EMT-related genes are found to play important roles in Chonglou against LUAD.

**FIGURE 8:**
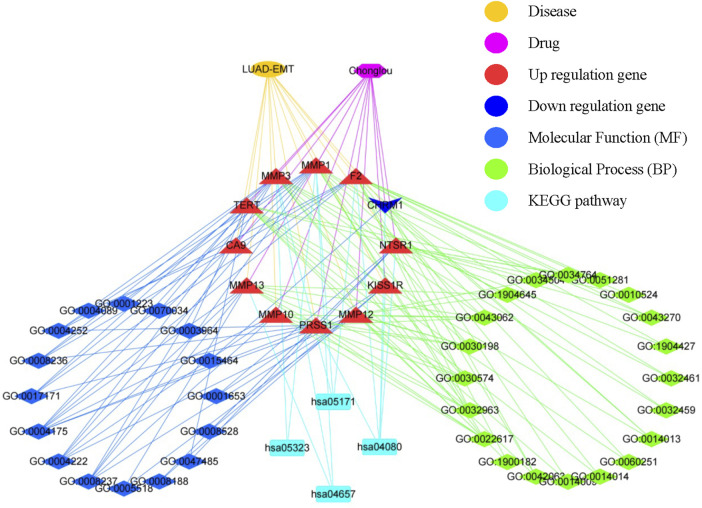
Interaction network showing the correlation between disease, drug, core biotargets, molecular function, biological process, and signaling pathways of EMT-LUAD-Chonglou related genes.

In order to explore the biological pathways playing important roles in Chonglou against LUAD, the 12 intersective genes obtained by STRING analysis mentioned above were submitted for GO and KEGG enrichment analysis. The results indicated that Chonglou active ingredients mainly affected the BPs, including extracellular matrix disassembly, collagen metabolic process, collagen catabolic process, extracellular matrix organization, extracellular structure organization, and response to amyloid-beta. As for the MFs, Chonglou active ingredients mainly affected the endopeptidase activity, metalloendopeptidase activity, metallopeptidase activity, serine-type endopeptidase activity, serine-type peptidase activity, and serine hydrolase activity and neuropeptide receptor activity. Furthermore, in the KEGG pathway analysis four pathways were identified, including neuroactive ligand-receptor interaction, IL-7 signaling pathway, coronavirus disease-COVID-19, and rheumatoid arthritis (Figure 7E). Moreover, the bioinformatics and network pharmacology were constructed, the results highlighted that extracellular matrix and anti-inflammatory were the key target/pathway of EMT-LUAD-Chonglou related genes (Figure 8).

### The active ingredients of Chonglou binds to the five core targets strongly

**FIGURE 9:**
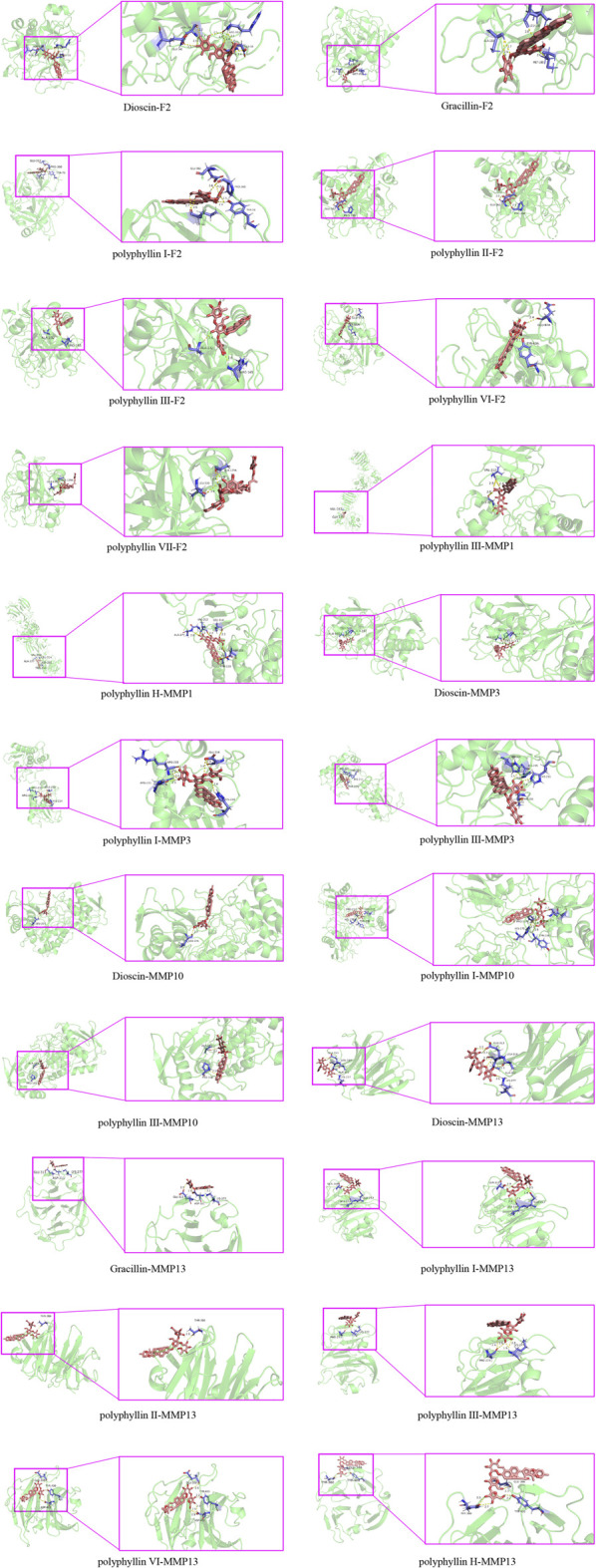
Schematic diagrams of the molecular dockings between the active ingredients of Chonglou and the hub genes.

Molecular docking models can predict the binding mode and binding affinity between the molecules to account for the differences in compound activities. To explore the potential bindings between the Chonglou active ingredients and the five core targets mentioned above, the molecular docking analysis was performed. The results revealed that the Chonglou active ingredients had strong binding abilities with the core targets, which showed that the binding energies between them were all less than −5 kcal mol^−1^ (Table 4). The docking results were visualized and the positions of the small molecules to form the hydrogen bonds with amino acid residues were marked on the map (Figure 9). Taken together, these bond interactions indicated that the Chonglou active ingredients had strong binding activities with the five core proteins.

**TABLE 4:** Molecular docking results between the active ingredients of Chonglou and the hub genes.

Active ingredients	Compound cid	F2 (kcal·mol-1)	MMP1 (kcal·mol-1)	MMP3 (kcal·mol-1)	MMP10 (kcal·mol-1)	MMP13 (kcal·mol-1)
Dioscin	119245	−10.68	—	−20.6	−11.45	−7.74
Gracillin	159861	−10.09	—	—	—	−9.21
polyphyllin I	71571451	−11.17	—	−17.94	−16.25	−7.95
polyphyllin II	46200821	−10.52	—	—	—	−6.32
polyphyllin III	101377611	−6.34	−8.37	−14.36	−8.28	−5.6
polyphyllin Ⅵ	10417550	−11.27	—	—	—	−9.28
polyphyllin Ⅶ	71307572	−5.44	—	—	—	—
polyphyllin H	101615586	—	−13.02	—	—	−10.99

### The active ingredients of Chonglou inhibits human lung adenocarcinoma cell proliferation

**FIGURE 10:**
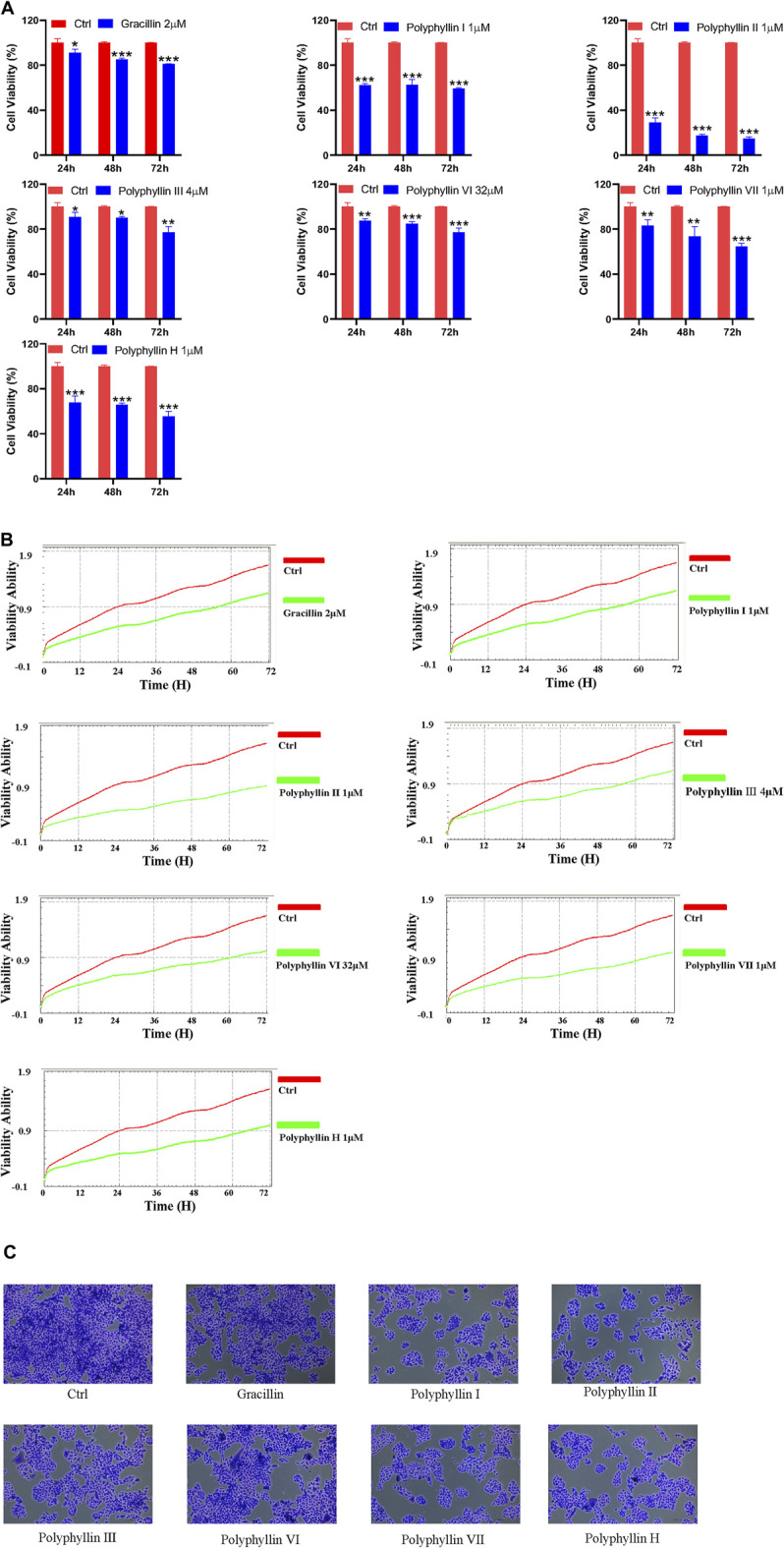
(Continued).

The active ingredient of Chonglou inhibited the cell viability and cell proliferation of lung adenocarcinoma cells. In the CCK-8 assay, the active ingredient of Chonglou significantly reduced the viability of lung adenocarcinoma cells intime-dependent manner, and subsequent observation of cell morphology under crystalline sub-staining revealed that the active ingredient of Chonglou significantly reduced the number of cell compared to untreated control cells. Furthermore, the viability of PC-9 cells was measured by xCELLigence RTCA DP system significantly inhibited the cell viability of lung adenocarcinoma cells in a time-dependent manner (Figure 10).

### The active ingredients of Chonglou inhibits human lung adenocarcinoma cell migration and invasion

**FIGURE 11:**
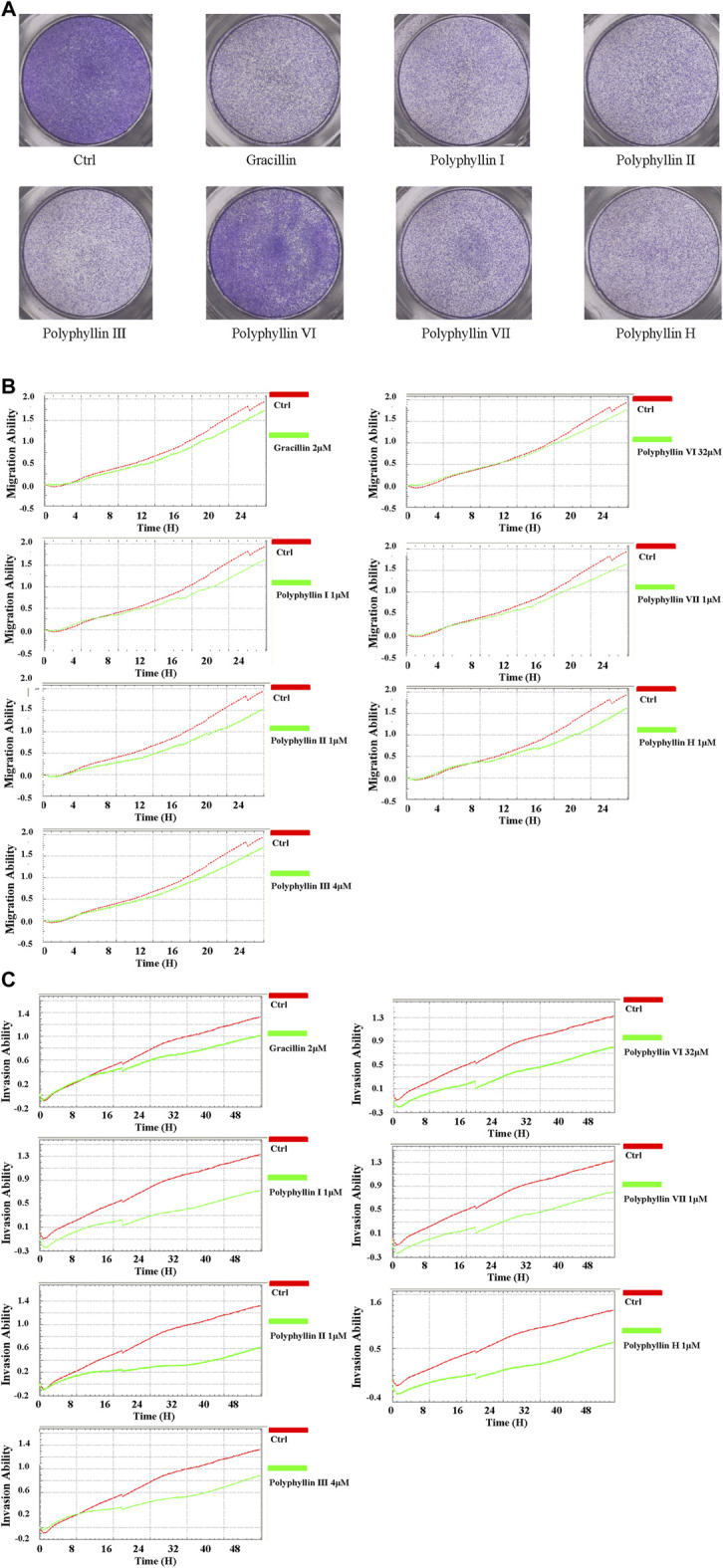
(Continued).

The Transwell migration examination and Real time migration and invasion assay were performed to investigate whether the eight active components of Chonglou affect the metastasis of human lung adenocarcinoma cells. Firstly, it was found that Gracillin, polyphyllin I, polyphyllin II, polyphyllin Ⅲ, polyphyllin Ⅵ, polyphyllin Ⅶ and polyphyllin H inhibited the migration and invasion of tumor cells compared with the control group, and it was found that the inhibition of PC-9 cell migration ability was time-dependent (0–24 h) (Figure 11).

## Discussion

LUAD is the predominant and the most lethal histopathological type of lung cancers, which ranks one of the most common carcinoma-related death (Uprety et al., 2020). Although significant progresses have made in target therapy and immunotherapy, the overall survival of LUAD patients is still poor due to the metastases (Shih et al., 2020). EMT has been recognized to play crucial role in the metastatic dissemination of cancers, including LUAD (Popper, 2016; Sung et al., 2016). Many active ingredients of Chonglou, one of the herbal medicines, have been reported to effectively control LUAD in the clinical practice (He et al., 2020; Teng et al., 2020).

During the more than 20 years that China proposed the task of modernising Chinese medicine, the modernization and globalization of traditional Chinese medicine (TCM) have gradually become an inevitable trend. With the modern interpretation and development of TCM by various new technologies such as big data, artificial intelligence and cloud computing, the inheritance and innovation of TCM, interdisciplinary and multidisciplinary integration and international cooperation are gradually being realized, which will help promote the further development of the inheritance and innovation of TCM, the development of national governance system and capacity, and the improvement of the quality of life of the global population.

To facilitate the clinical translation and application, the EMT-related mechanisms of Chonglou active ingredients in LUAD treatment was explored with a comprehensive bioinformatics analysis in this study, and 166 EMT-LUAD related genes were identified, including 159 up-regulated genes and 7 down-regulated genes. The multivariate Cox proportional hazards regression model revealed that the AUC values of the ROC curves for the 1, 3, and 5-year overall were .729, .7, and .679, indicating this model established based on the EMT-LUAD-related genes is well applicable to predict the prognosis of LUAD patients and further illustrates the implication of EMT-LUAD-related genes in LUAD patients.

After a comprehensive analysis, the clinical characteristics, immune cell infiltrations, immune cell subsets and checkpoint inhibitors were identified to be significantly different in the high- and the low-risk group, indicating the close association between the immune system with the risk scores of the EMT-LUAD related genes with independent significant prognostic value of all included LUAD patients. This also implied that the immune microenvironment in the LUAD patients is regulated by the EMT-LUAD related genes, which is essential for LUAD development, thus to generate candidate predictive biomarkers for the response to immunotherapies and guide the identification of new immunotherapeutic interventions (Gajewski et al., 2013; Hinshaw and Shevde, 2019).

By using the PPI network analysis, 12 EMT-LUAD-Chonglou related genes and five core drug targets (F2, MMP1, MMP3, MMP10, and MMP13) were identified. GO and KEGG enrichment analysis revealed that the effective components of Chonglou may play significant roles in LUAD treatment by regulating the Biological Process (BP) of extracellular matrix, collagen metabolism, extracellular structure, inflammatory response and related signaling pathways, including extracellular matrix disassembly, collagen metabolic process, collagen catabolic process, extracellular matrix organization, extracellular structure organization, and IL-7 signaling pathway. Since the cellular matrix, inflammatory responses and collagen are important in the tumor microenvironment (TME), which is a complex and continuously evolving environment including stromal cells, fibroblasts, endothelial cells and innate and adaptive immune cells with diverse capacities to control the consequences of tumorigenesis (Quail and Joyce, 2013; Hinshaw and Shevde, 2019). These findings will help to clarify the mechanism of Chonglou active ingredients in controlling LUAD development, provide possible drug targets and therapeutics for the subsequent treatment.

When further molecular docking was carried out, we found that there was an effective binding affinity between the active ingredient of Chonglou and the key proteins of LUAD including F2, MMP1 with all the values of kcal·mol-1 less than −5, which suggest that the active ingredients of Chonglou could bind stably to the protein and may act as a the promising drug targets in LUAD treatment. It has been reported that F2 is an important factor for lung cancer cell-induced platelet aggregation (Heinmöller et al., 1996), and the platelet aggregation is directly related to the metastatic ability of cancer cells (Tang and Honn, 1994).

MMPs can be divided six subfamilies, of which MMP1 and MMP13 are collagenases and MMP3 and MMP10 are stromelysins, which have long been associated with tumor invasion, metastasis and angiogenesis (Das et al., 2021). MMP1 plays a key role in the proliferation and migration of cancer cells, as well as the angiogenesis of cancer (Wang et al., 2011). Upregulation of MMP1 contributes to the malignant development in LUAD patients (Schütz et al., 2015). MMP1 is a vital therapeutic target for drug development in LUAD (Saito et al., 2018). MMP3 has been reported to play an important role in the metastasis of LUAD (Yang et al., 2019). High expression of MMP13 also increases the risk of brain metastases in LUAD patients (Shih et al., 2020). Therefore, our results of enrichment analysis and molecular docking illustrated that the EMT-LUAD-Chonglou related genes may be the effective drug targets for the treatment of metastatic LUAD with the active ingredients of Chonglou, which provides the promising candidates for the clinical treatment of metastatic LUAD.

Proving that the active ingredient of Chonglou has anti-tumor effect is the basis of our research, so the active ingredient of Chonglou inhibits the proliferation, migration and invasion of lung adenocarcinoma cells has been studied.

Real-time monitoring with xCELLigence RTCA DP system showed that the active components of Chonglou could inhibit cell viability, and the migration ability of PC-9 cells was hindered by the active components of Chonglou, which was confirmed by transwell and further verified by real-time monitoring with xCELLigence RTCA DP system. At the same time, real-time monitoring with xCELLigence RTCA DP system also showed similar results of invasion ability of active components of Chonglou to PC-9 cells. All in all, these findings indicate that the active components of Chonglou inhibit the occurrence and metastasis of lung adenocarcinoma *in vitro*, and verify that the establishment of our target can be traced.

In conclusion, this study revealed five key EMT-related drug targets among the active ingredients of Chonglou that have relevance in LUAD treatment, including F2, MMP1, MMP3, MMP10, and MMP13, which are associated with the extracellular matrix, collagen metabolism and inflammatory responses. These findings will direct future preclinical research and facilitate the clinical translation in LUAD management.

## Data Availability

The original contributions presented in the study are included in the article/Supplementary Material, further inquiries can be directed to the corresponding authors.
